# DTI and Myelin Plasticity in Bipolar Disorder: Integrating Neuroimaging and Neuropathological Findings

**DOI:** 10.3389/fpsyt.2016.00021

**Published:** 2016-03-01

**Authors:** Marcella Bellani, Filippo Boschello, Giuseppe Delvecchio, Nicola Dusi, Carlo Alfredo Altamura, Mirella Ruggeri, Paolo Brambilla

**Affiliations:** ^1^Section of Psychiatry, Azienda Ospedaliera Universitaria Integrata Verona, Verona, Italy; ^2^Section of Psychiatry, University of Verona, Verona, Italy; ^3^IRCCS “E. Medea” Scientific Institute, Italy; ^4^Department of Neurosciences and Mental Health, Ospedale Maggiore Policlinico, Fondazione IRCCS Ca’ Granda, University of Milan, Milan, Italy; ^5^Department of Psychiatry and Behavioural Neurosciences, University of Texas at Houston, Houston, TX, USA

**Keywords:** bipolar disorder, diffusion tensor imaging, myelin plasticity, oligodendrocyte, white matter disruption, connectivity

## Abstract

Bipolar disorder (BD) is a major psychiatric illness with a chronic recurrent course, ranked among the worldwide leading disabling diseases. Its pathophysiology is still not completely understood and findings are still inconclusive, though a great interest on the topic has been constantly raised by magnetic resonance imaging, genetic and neuropathological studies. In recent years, diffusion tensor imaging (DTI) investigations have prompted interest in the key role of white matter (WM) abnormalities in BD. In this report, we summarize and comment recent findings from DTI studies in BD, reporting fractional anisotropy as putative measure of WM integrity, as well as recent data from neuropathological studies focusing on oligodendrocyte involvement in WM alterations in BD. DTI research indicates that BD is most commonly associated with a WM disruption within the fronto-limbic network, which may be accompanied by other WM changes spread throughout temporal and parietal regions. Neuropathological studies, mainly focused on the fronto-limbic network, have repeatedly shown a loss in cortical and subcortical oligodendrocyte cell count, although an increased subcortical oligodendrocyte density has been also documented suggesting a putative role in remyelination processes for oligodendrocytes in BD. According to our review, a greater integration between DTI and morphological findings is needed in order to elucidate processes affecting WM, either glial loss or myelin plasticity, on the basis of a more targeted research in BD.

## Introduction

Bipolar disorder (BD) is a major psychiatric illness that affects 1% of the population and has its onset during adolescence or early adulthood (1, 2). It significantly decreases the quality of life of patients and caregivers and increases the burden of health and social care services (3).

Since 90s, a growing interest on the neurobiological underpinnings of BD has been object of several magnetic resonance imaging (MRI) studies that, so far, have repeatedly reported diffuse white matter (WM) hyperintensities at T2-weighted sequences, spread out over subcortical, periventricular and callosal WM (4, 5). BD patients are 2.5 times more likely than healthy controls and 2 times more likely than depressed patients to show these neurobiological profiles, which have been therefore considered as a putative biological correlate of BD (5–7). Pieces of evidence suggested an association between more severe WM hyperintensities and poorer outcome measures, such as increased clinical severity (8), greater number of hospitalizations, poorer response to treatments (9) and higher suicide risk (10). Nevertheless, similar patterns of WM alterations did not seem to be specific to BD, as they have been found in neuropathological processes, such as cerebrovascular damage, astrocytic gliosis and demyelination processes (6).

A weakness of structural MRI in studying WM is the limited contrast detection in WM tracts, while diffusion tensor imaging (DTI) is uniquely sensitive to WM microstructure analysis including axonal coherence, fiber density and myelin integity (11, 12). Since 2000s WM abnormalities in BD have been investigated using this imaging technique, which consists of a specific extension of MRI technology and allows a more precise identification of subtle WM derangements (13–15). Basically, DTI applies two indices for the study of WM’s physical integrity: the fractional anisotropy (FA) and the apparent diffusion coefficient (ADC). FA measures the anisotropic diffusion of water molecules and ranges between 0 and 1. When water molecules diffuse along the neural fibers on their longitudinal axes, due to their directional movement constraint by thick myelin sheaths, FA is 1, whereas when water molecules diffuse toward random directions, following their Brownian motion, FA is 0. ADC is an index of the rate of water diffusion in cerebral tissues: higher values of ADC indicate less restricted diffusion; conversely, lower values of ADC point to the presence of organized fibers impeding water diffusion (16). To date, overall DTI findings in BD have reported either lower, higher or no difference in FA between patients and healthy controls (12, 15, 17). Inconsistency across findings is supposed to reflect differences among DTI studies in data acquisition protocols as well as in patient characteristics (18). Most studies involve heterogeneous samples of BD patients combining subjects in different mood states (19), which could have different neural network activation correlates (20) and different WM DTI patterns (18, 21). Moreover, BD patients’ samples may differ for medication status (22), which could be considered as a relevant variable for interpreting findings. Indeed, lithium seems to act as a promyelinating factor (23, 24), whereas first and second generation antipsychotics are likely to impact on WM integrity, despite their effects vary markedly across studies ranging from promyelinating actions (24, 25) to impaired WM integrity (26).

The disruption of neural connectivity due to myelin sheath’s degradation, whose etiology is not yet fully understood, is supposed to play a compelling role in the neurobiology of BD (7, 27). The molecular and cellular correlates of these structural MR and DTI findings have not been completely explained yet, although the role of glial cells has been implicated in the disruption of neural connectivity (28). In the human brain tissue, glial cells include oligodendrocytes (35%), oligodendrocytes progenitor cells (OPCs) (5%), astrocytes (45%) and microglia (5%) (24). A great amount of oligodendrocytes arise from the differentiation of OPCs till the early postnatal period, yet to a lesser extent OPCs persist in generating oligodendrocytes during adulthood (29). Adult born oligodendrocytes might be involved in *de novo* myelination of previously naked or not completed myelinated axons, as well as in myelin remodeling that allows changes in existing myelin membrane sheaths in the central nervous system (CNS) (30, 31). Many DTI studies, genome wide association studies and postmortem studies have demonstrated that BD patients showed a reduction in myelin content in the CNS, especially in subcortical regions, along with aberrant expression of myelin-related genes and a reduction in oligodendrocyte cells’ count (32–34). These findings are pointing to the role of glial cells’ abnormalities in the disruption of WM connectivity in BD. However, a clear correlation between WM alterations in DTI studies and changes in glial cells’ population in neuropathological studies is still lacking.

In this review, we address the issue of WM alterations in BD, based on the data from the most recent DTI studies, and we focus on oligodendrocyte involvement in WM alterations in BD, emphasizing the role of myelin plasticity on the basis of the available lines of evidence.

## Materials and Methods

We carried out a literature search of published studies on the following databases: National Centre for Biotechnology Information (NCBI) PubMed (MEDLINE) and Google Scholar. We took into account published papers from August 2009 to August 2015 focusing on the following key words: bipolar disorder, diffusion tensor imaging, white matter, myelin plasticity and oligodendrocytes. The reference lists of the included studies were then searched for additional studies.

Firstly, we only considered studies published in English with a patient population of 18–65 years old affected by BD, according to DSM-IV or ICD-10 criteria, where a DTI scan was performed. We limited our analysis to FA values, as other measures of diffusivity, while potentially informative about WM impairments, were inconsistently applied across DTI studies. We included DTI studies applying either a region of interest (ROI)-based analysis or a whole brain analysis. Among whole brain DTI studies we used only voxel-based analysis (VBA) and tract-based spatial statistics (TBSS) analysis, excluding DTI tractography studies given that methodological issues are deemed to undermine a direct comparison between tractography and VBA approaches (35). Indeed, VBA images are smoothed and statistical thresholds are applied to generate a statistical map, where differences of voxel-wise mean values between groups are shown at a local level in brain regions, instead of tractography where DTI metric values are averaged along a 3-D ROI and local differences in specific regions of the reconstructed tract could be lost (35).The search of the literature revealed 88 publications suitable for the inclusion in the present paper, of these 13 were literature reviews and 2 were meta-analyses (see Table 1).

**Table 1:** **Reviews and meta-analyses of diffusion tensor imaging studies investigating WM in patients with bipolar disorder**.

Reference	Metodology	Subjects	Main findings in BD patients
Teipel et al. (36)	Review of ROI studies and VBA studies	−	↓ FA in fronto-occipital and callosal connections, SLF, UF. ↓ FA right-sided in WM close to the PHG and sgACC
Dell’Osso et al. (37)	Review of ROI studies and VBA studies	−	↓ FA in WM of FC and OC, cingulum bundle, CC, internal capsule and FOF. ↓ FA right-sided in WM close to the PHG and sgACC. ↓ FA in ACR. ↑ FA in corticopontine/corticospinal tracts, SLF, TR
Marlinge et al. (38)	Review of ROI studies and VBA studies	−	↓ FA in CC, ACR, internal capsule and FOF. ↓ FA in WM close to right PHG (SLF, IFOF, ILF, PTR) and in WM close to right sgACC and right ACC
Hahn et al. (3)	Review of ROI studies and VBA studies	271 BD pts vs. 108 HC	↓ FA in CC ventral part in late-life BD. No studies on WM alterations in late-onset BD
Shizukuishi et al. (39)	Review of ROI studies and TBSS studies	−	↓ FA in PF WM and ↑ FA in the genu of CC. ↑ FA in left UF and ↓ FA in right UF
Nortje et al. (40)	Review and meta-analysis of VBA studies and TBSS studies	252 BD pts vs. 256 HC	↓ FA in a right posterior tempo-parietal WM cluster and in two left cingulate WM clusters
Hafeman et al. (41)	Review of ROI studies and VBA studies	430 BD pts vs. 402 HC	↓ FA (especially in depressed patients) in CC, UF, SLF, anterior TR, ACB. Medicated BD pts more similar to HC than their unmedicated counterparts
Vederine et al. (42)	Review and meta-analysis of VBA studies	289 BD pts vs. 279 HC	↓ FA in a WM cluster close to right PHG (SLF, IFOF, ILF, posterior TR) and a WM cluster close right ACC and right sgACC
Bellani and Brambilla (18)	Review of ROI studies, VBA studies, TBSS studies	429 BD pts vs. 436 HC	↓ FA in SLF, FOF and CC
Mahon et al. (43)	Review of ROI studies, VBA studies, TBSS studies	524 BD pts vs. 561 HC	↓ FA along the UF and other WM tracts subserving both the OFC and the ACC as well as in TR fibers and SLF
Heng et al. (15)	Review of ROI studies, VBA studies, TBSS studies	465 BD pts vs. 480 HC	↓ FA in PF WM. ↓ FA in projection, associative and commissural WM fibers
Palaniyappan et al. (44)	Review of ROI studies, VBA studies, TBSS studies	−	↓ FA in PF WM, ACB, temporal WM. ↑ FA in genu of CC. ↓ FA in UF, ACR, cingulate–amygdala–hippocampal connections
Agarwal et al. (6)	Review of ROI studies and VBA studies	−	↓ FA in PF WM, CC and internal capsule

*BD, bipolar disorder; WM, white matter; HC, healthy controls; DTI, diffusion tensor imaging; ROI, region of interest; VBA, voxel-based analysis; TBSS, tract-based spatial statistics; FA, fractional anisotropy; pts, patients; CC, corpus callosum; SLF, superior longitudinal fasciculus; UF, uncinated fasciculus; PHG, parahippocampal gyrus; sgACC, subgenual Anterior Cingulate Cortex; ACC, anterior cingulate cortex; FC, frontal cortex; OC, occipital cortex; FOF, fronto-occipital fasciculus; ACR, anterior corona radiata, TR, thalamic radiation; IFOF, inferior fronto-occipital fasciculus; ILF, inferior longitudinal fasciculus; PTR, posterior thalamic radiation; PF, prefrontal; OFC, orbitofrontal cortex; OF, orbitofrontal; ACR, anterior corona radiata, ACB, anterior cingulum bundle*.

Secondly, we focused on studies published in English with a patient population of 18–65 years old affected by BD, according to DSM-IV or ICD-10 criteria, where the oligodendrocyte involvement was specifically addressed at a neuropathological level. We included studies focused on perineuronal and myelinating oligodendrocytes because oligodendrocytes exist both in GM and WM and alterations in GM glial organization are thought to be related to WM changes (43). We excluded and considered unfocused all the studies investigating glial cells without considering oligodendrocyte density (OD) in BD patients and controls. Eight studies identified by the literature search were judged to fulfill these inclusion criteria (see Table 2).

**Table 2:** **Neuropathological postmortem studies focusing on oligodendrocyte involvement in bipolar disorder**.

Reference	Methodology	Subjects	Age, years (mean ± SD)	Sex ratio M/F	Main findings
Hercher et al. (28)	Neuropathological postmortem assessment of DLPC WM in SZ, BD, and HC (2-D cell counting technique)	20 SZ vs. 20 BD vs. 20 HC	SZ 44.7 (±6.9), BD 47.4 (±0.7), HC 45 (±6.5)	SZ 13/7, BD 8/12, HC 14/6	↑ OD in BD vs. HC. No differences in SZ vs. HC. No differences in AD among the groups just a trend toward ↓ AD in SZ vs. HC
Savitz et al. (45)	Review of postmortem studies on mPFC neuronal density and glial cell density in BD and HC	−	−	−	3-D cell counting studies:↓ glial cell density (predominantly ↓ OD) in BD vs. HC. Unclear if ↓ ND in BD vs. HC
Haroutunian et al. (46)	Review of postmortem studies and genetic studies on myelin and oligodendrocyte involvement in SZ, BD, and MDD	−	−	−	↓ cortical OD and myelin gene disruption in SZ, BD and MDD vs. HC. ↓ subcortical OD in BD and MDD vs. SZ
Williams et al. (47)	Neuropathological postmortem assessment in sgACC and genu of CC among SZ, BD, MDD, and HC (2-D cell counting technique)	10 SZ vs. 15 BD vs. 20 MDD vs. 19 HC	SZ 65.5 (±2.3), BD 56.1 (±5.2), MDD 47.6 (±3.1), HC 65.5 (±2.3)	SZ 5/5, BD 6/9, MDD 7/13, HC 11/8	↓ AD in sgACC and anterior CC WM in SZ vs. HC. No changes in OD among different groups in sgACC and anterior CC WM
Gos et al. (48)	Neuropathological postmortem assessment of AD and OD in HPC (alveus and CA1 pyramidal layer) among MDD, BD, and HC (2-D cell counting technique)	6 BD vs. 9 MDD vs. 13 HC	BD 55.7 (±13.3), MDD 49.6 (±11), HC 55.3 (±12.3)	BD 3/3, MDD 2/7, HC 7/5	Bilateral ↓ AD in CA1 pyramidal layer in MDD and BD vs. HC. ↓ OD in left alveus only in BD vs. HC
Hayashi et al. (49)	Neuropathological postmortem assessment of PFC and ITC GM to examine OD among SZ, BD, and HC (3-D cell counting technique)	10 PFC SZ vs. 12 PFC BD vs. 12 PFC HC 11 ITC SZ vs. 11 ITC BD vs. 11 ITC HC	PFC SZ 43 (±14), BD 41 (±11), HC 48 (±11) ITC SZ 45 (±14), BD 41 (±12), HC 50 (±10)	PFC: SZ 7/3, BD 7/5, HC 8/4 ITC: SZ 6/5, BD 6/5, HC 7/5	↓ OD in PFC GM of BD vs. SZ and HC. ↓ ID in ITC GM of BD and SZ vs. HC
Mahon et al. (43)	Review of postmortem neuroimaging and genetic studies on WM in BD	−	−	−	3-D cell counting studies: ↓ cortical OD in sgACC and DLPC in BD vs. HC. Disruption in myelin and oligodendrocyte-related genes expression in BD
Vostrikov et al. (50)	Neuropathological postmortem assessment of OD related to age in PFC among SZ, MDD, BD, and HC (2-D cell counting technique)	15 SZ vs. 15 BD vs. 15 MDD vs. 15 HC	SZ 44.5 (±13.1), BD 42.3 (±11.7), MDD 46.5 (±9.3), HC 48.1 (±10.7)	SZ 9/6, BD 9/6, MDD 9/6, HC 9/6	↓ OD in young BD vs. HC. Age-related ↑ OD in HC, but not in SZ, MDD, and BD

*SZ, schizophrenia; BD, bipolar disorder; MDD, major depressive disorder; HC, healthy controls; pts, patients; GM, gray matter; WM, white matter; DLPFC, dorso lateral prefrontal cortex; OD, oligodencrocyte density; ID, interneuron density; AD, astrocyte density; ND, neuronal density; sgACC, subgenual Anterior Cingulate Cortex; CC, corpus callosum; HPC, hippocampus; ITC, inferior temporal cortex*.

## Results and Discussion

Several structural MRI studies, using both ROI analysis and VBA, indicated that WM hyperintensities, especially in the prefrontal and frontal WM, were associated with BD compared with healthy controls (4, 43). These WM abnormalities’ observations have been replicated in studies on first-episode BD patients (51) and adolescents with BD (52, 53). All together, these studies suggested that, besides confounders as brain aging and medical illnesses, other processes should be involved in the pathophysiology of BD.

Diffusion tensor imaging provides a more sensitive tool, compared to structural MRI, to investigate WM integrity and microstructure (11, 12). In the following paragraphs, DTI studies accordingly to the different methodological approaches – ROI analysis, VBA and TBSS analysis – will be briefly debated.

### ROI-Based Analysis

Most of DTI studies based on ROI approach showed lower FA among BD patients compared with healthy controls, mainly in WM tracts in prefrontal areas, anterior cingulum, callosal areas, and limbic-striatal areas (54–56). These abnormalities, particularly in deep prefrontal WM, have been reported since the first episode of mania (14). In support of these findings, a review of ROI-based studies has recently confirmed a loss of bundle coherence and alignment of WM fibers in the following areas in BD: deep WM in frontal and occipital lobes, anterior cingulum, corpus callosum (CC), anterior corona radiata and internal capsule (38). Though consistent and replicated, these results might be limited by a selection bias, as ROI-based approach is hypothesis-driven and provides information only about selected brain regions.

### Voxel-Based Analysis

Voxel-based analysis allows DTI data analysis over the whole brain volume without *a priori* hypotheses on specific regions of interest, thus overcoming possible selection bias from the ROI approach.

The current findings from VBA-DTI studies suggest the existence of diffuse WM microstructural abnormalities as BD’s trait feature, raising the intriguing notion of a disconnected framework within fronto-limbic areas as well as between fronto-limbic and temporal, parietal and occipital lobes. A recent meta-analysis (40) showed decreased FA in all major classes of WM tracts (i.e., commissural, association and projection), with more robust findings on Bipolar Type 1 vs. Bipolar Type 2 patients. VBA data displayed significant clusters of decreased FA within right temporo-parietal WM, involving both inferior fronto-occipital fasciculus (IFOF) and inferior longitudinal fasciculus (ILF), left cingulum and left anterior cingulate. These findings suggest a wide alteration in the WM of CNS in BD, which involves not only an anterior fronto-limbic pathway but also fibers connecting temporal and parietal cortices. Consistent with these observations, a recent integrated structural MRI/DTI study (57) found a global reduction of WM volume and WM clusters of decreased FA in BD patients, ranging from prefrontal WM to the splenium of CC and posterior cingulum bundle. Additionally, the authors also investigated whether pharmacological treatment could have influenced their findings and they observed no significant association between lithium medication and any DTI measures, while total GM volume was increased with significant clusters in temporal lobes in patients treated with lithium. Moreover, a meta-analysis of VBA-DTI studies in BD (38) found two consistent clusters of FA and mean diffusivity alterations both of them located in areas relevant for emotional processing in the right hemisphere. The first cluster was close to the right parahippocampal gyrus in a WM area crossed by the superior longitudinal fasciculus (SLF), ILF, IFOF and posterior thalamic radiations, while the second one was close to the right anterior cingulate cortex (ACC) and the right subgenual cingulate cortex (sgACC). These findings prompted the authors to speculate that a pattern of disrupted WM connectivity may underlie the abnormal emotional processing in BD patients compared with healthy controls. It is worth highlighting that a disruption of SLF might contribute to fronto-temporal disconnection, which could be relevant to emotional cognitive modulation and inhibition, whereas the disruption of ILF and IFOF could underlie facial emotion recognition deficits, proposed as a potential endophenotype for BD (58, 59). Furthermore, both ACC and sgACC are thought to be involved in identification of emotionally salient stimuli and WM diffusion abnormalities, clustered close to these areas, have been associated with dysfunctional emotional processes in BD (60). Indeed, a disrupted WM connectivity between these regions and a dorsal cognitive network [dorsolateral prefrontal cortex (DLPFC), dorsal cingulate cortex, precuneus, cuneus] is supposed to play a role in over reactivity of ACC and sgACC during emotional processing in bipolar patients (61).

White matter microstructure changes have been also regarded as a possible DTI endophenotypic marker in BD. Interestingly, a VBA-DTI study comparing individuals with BD, their ultra-risk (UR) relatives and healthy controls found no significant FA differences in UR vs. healthy controls (62). However, the *post hoc* analysis, including the density of illness within families, revealed a reduction in FA associated with increasing genetic liability in prefrontal WM, SLF, CC and uncinate fasciculus. Furthermore, a lower FA in SLF has been reported in children at risk of BD in a study comparing children at risk of BD with a sample of unaffected children (63).

Eventually, the notion of early WM markers of vulnerability for BD has been recently strengthened by a review of DTI studies in BD children and adolescents (2). In keeping with VBA-DTI findings in adults with BD, the study reported that BD children and adolescents showed a pattern of decreased FA both in fronto-temporal intrahemispheric WM (prefrontal WM, cingulum bundle, WM bundles to dorsal frontal regions, basal ganglia, thalamus and posterior association cortices) and fronto-temporal interhemispheric WM (anterior CC).

### Tract-Based Spatial Statistics Analysis

Tract-based spatial statistics analysis is a whole brain analysis of DTI data, which focuses on the centers of all fiber bundles, improving the probability that the given space voxels contain uniform data from the same WM tract (the most compact WM skeleton). TBSS approach overcomes the misinterpretation of crossing fibers areas hindering the reliability of traditional voxel-wise analysis (64).

Significant widespread decreases in FA have been predominantly reported so far by TBSS DTI studies among BD patients compared with healthy controls with WM changes encompassing all major WM tracts (projection, association, callosal and limbic fibers). Indeed, in a recent published meta-analysis of TBSS studies, Nortje and colleagues (40) showed a decreased FA in all main classes of WM tracts, though the pattern of mostly involved bundles was different among independent studies. Consistent with these results, a TBSS study, employing a group of bipolar I and II patients in comparison with a group of healthy controls, reported in BD patients a significant lower FA in CC, cingulum bundles, fornices, SLF, ILF, fronto-occipital fasciculus, thalamic radiation and uncinate fasciculus (65). These widespread WM diffusion abnormalities, including fronto-temporal and ventral striatal regions, were marked by significant differences in diffusivity measures other than FA, leading the authors to conclude that dysmyelination and demyelination rather than axonal loss are implicated in BD (66, 67). Providing further support to these lines of evidence, a TBSS study considering 40 inpatients with bipolar depression vs. 21 healthy controls showed a reduced FA in the following WM areas: CC, cingulum, corona radiata and SLF (68). The authors considered the disrupted connectivity evidenced in interhemispheric connections as well as in frontal, parietal and fronto-occipital connections as the biological underpinning of cognitive and emotional deficits of bipolar depression. Notably, in this study, lower FA values were coupled with higher radial diffusivity and mean diffusivity with no differences in parallel diffusivity, consistently with those findings showing a disruption in myelin sheaths without axonal loss (69).

Finally, DTI measures have been also investigated as biomarkers of treatment response, even though robust evidence on this subject is still missing with studies focusing mostly on major depressive disorder (70, 71). To the best of our knowledge, the role of WM microstructure in predicting treatment efficacy in BD has been investigated in just one TBSS DTI study (72). Authors revealed a significant association between poor response to chronotherapeutic treatment in bipolar depression and increased radial diffusivity and mean diffusivity in WM tracts involved in cognitive and emotional deficits (CC, cingulum bundle, IFOF, SLF, corona radiata and anterior thalamic radiation).

Unfortunately, DTI measures, regardless of which approach is applied, are relatively non-specific and do not clearly differentiate between axon and myelin-based abnormalities (73). As outlined in a recently published study (74), DTI quantifies diffusion of water molecules and water exists in both intracellular and extracellular spaces (with exchange between two). Thus, the authors applied a more refined MRI approach, using magnetization transfer ratio, in order to study myelin content, and diffusion tensor spectroscopy, in order to study *N*-acetylaspartate diffusion within axons. Their results showed reduced myelin content but no changes in intra-axonal geometry, considered as a proxy of axonal integrity, in patients with BD vs. healthy controls. Furthermore, axonal demyelination is just one of several possible factors impacting on FA values, including bundle coherence, crossing fibers, water concentration and neuronal loss (13). Therefore, findings of abnormal FA in either direction that are not supported or followed up by neuropathological postmortem investigations should be interpreted with caution (43).

### Myelin Plasticity

In humans, developmental myelination is a long-lasting process continuing at least until the fourth decade of life (75, 76) and proceeding in predictable spatial and temporal patterns (i.e., thicker axons are myelinated before thinner axons, brain regions devoted to more basic and homeostatic functions, such as brainstem, are myelinated before high level integrative areas, such as cortical association fibers) (77). During and even after the developmental myelination, mammalian CNS myelin has shown to be plastic and sensitive to experience dependent structural changes: this feature is referred to as myelin plasticity (78).

So far, postmortem studies of individuals with BD have shown sparse and contradictory findings about WM and GM changes in number, size and density of different glial cell populations (43, 46, 49). As regards the oligodendrocytes involvement, evidence from several morphometric postmortem studies, identified in our literature search, pointed out that BD patients might be affected by an oligodendrocyte cell loss in the same areas where DTI studies have reported WM diffusivity abnormalities. Indeed, lending support to this speculation, a review of postmortem neuropathological studies, focusing on medial prefrontal cortex (mPFC) network in BD patients, has recently reported a reduction in glial cells both in sgACC and DLPFC (45). Both perineuronal and myelinating oligodendroglia cells were the most clearly involved glial cell populations. Interestingly, the oligodendroglia cell loss in BD was correlated with a putative increase of neuronal density in sgACC, orbitofrontal cortex (OFC) and DLPFC due to dendritic atrophy (the great amount of GM consisting of neuropil). Furthermore, a neuropathological study (48) evaluating astro and oligodendroglial expression of SB100 (a glial marker protein), in CA1 pyramidal layer and the alveus of hippocampus, showed a decreased OD in BD compared with healthy controls in the left alveus. Congruent with these neuropathological observations, many genetic studies, which support the role of oligodendrocyte dysfunction in BD, reported a downregulation of key oligodendrocyte and myelination genes in BD compared with healthy controls (20, 24, 79, 80). Notably, Tkachev and colleagues (81) provided a strong evidence for a downregulation of key oligodendrocyte and myelination genes, including transcription factors that regulate these genes, in bipolar and schizophrenic patients compared with healthy controls, by using quantitative polymerase chain reaction and microarray analysis. A selective reduction in oligodendrocyte-related gene expression was noted, after controlling for confounding variables such as medication and alcohol abuse, pointing toward a cellular dysfunction or death. Even if there are not conclusive evidences, the mechanism underlying an oligodendroglia cell loss is likely to affect inhibitory GABAergic transmission: a GABAergic interneuron loss, which decreases the inhibitory feedback on glutamatergic neurons, may lead to excitotoxicity-induced dendritic atrophy and/or oligodendrocyte cell loss (82). As known, oligodendrocytes express AMPA and kainate-type glutamate receptors, therefore they are highly sensitive to excitotoxic damage from an excessive glutamate-mediated activation (83, 84).

However, in contrast to the evidence for an oligodedroglia cell loss, a recent study investigating the morphological basis underlying WM alterations located in DLPFC in bipolar and schizophrenia patients showed an increased OD in the BD group compared both with schizophrenia and healthy control subjects (28). It is worth noting that the increased OD in the subcortical WM of DLPFC was not correlated with higher myelin content, as evidenced by the increase of a marker protein located in oligodendrocyte cell bodies (2′3′ cyclic 3′phosphodiesterase) and no changes in a marker protein located in compact myelin (myelin basic protein). These findings are very intriguing as, along with the observation of an increased myelin peroxidation in the myelin fraction in BD patients (85), they strengthen the hypothesis that an increased OD could reflect a compensatory mechanism of repairing damage. In this perspective, the proliferation and differentiation of OPCs into mature oligodendrocytes could play a role in replacing damaged myelin throughout a remyelination process in the adult brain. With regard to the putative compensatory mechanism to restore damaged myelin, Bartzokis (24) has mainly shifted the emphasis from subcortical into intracortical myelination processes as key factor in better understanding BD’s pathophysiology. In the human brain development, during childhood the subcortical WM myelination would achieve a network of axons’ synchronization, connecting widely distributed brain regions, whereas throughout adulthood the intracortical myelination would make faster and more synchronized this transmission, by adding further myelin sheath. Intracortical myelin could compensate for a disruption in transmission along WM subcortical fibers, due to either subcortical myelin damages or subcortical remyelination processes which produce thinner myelin sheaths. These events could influence the quality of neurotransmission as the thickness of myelin sheaths is inversely correlated with speed of conduction and network synchronization (19). If an adequate intracortical myelin plasticity may initially compensate for subcortical transmission delays, during the course of the illness an increasing intracortical myelin disruption could undermine this compensation mechanism and thus give raise to an increasingly disrupted WM connectivity (24). At the onset of BD, subcortical myelin deficits seem to be more prominent than intracortical myelin deficits (86) but, during the course of the illness, significant intracortical oligodendrocyte deficits develop in BD (50) while cognitive deficits worsen (87, 88). Noteworthily, converging evidence of a decreased OD in the gray matter of DLFC and sgACC in BD, as compared to healthy controls, has been raised by several reviews of morphometric studies (43, 45), though not homogeneous in terms of cell counting technique applied (either 2-dimensional or 3-dimensional). Furthermore, a study (49) applying a 3-dimensional cell counting method confirmed the existence of a significantly decreased intracortical OD in the PFC of BD patients as compared not only to healthy controls but also to SZ patients, thus suggesting this neuropathological abnormality as more commonly associated to BD than SZ. Eventually, it is of interest to mention that another study (50) reported a reduction of OD in PFC of BD patients along with a loss of age-related increase in OD of either BD or SZ or MDD patients in layer VI of the PFC (BA 9) as compared to controls. These findings led the authors to postulate that common age-related process of OD increase is dysregulated in SZ and mood disorders. Taken together, these findings are consistent with a oligodendrocyte cell loss affecting the intracortical myelination within the fronto-limbic network in BD, whereas direct evidence from human neuropathological studies for the role of myelin plasticity in BD is still missing.

It should be noted that neuropathological results regarding WM alterations in BD are limited by several key factors listed/described as follows (89, 90): they are prominently focused on prefrontal WM and show heterogeneous and quite inconsistent findings; there are wide discrepancies between the 2-D vs. 3-D counting methods, given that caution must be taken particularly with 2-D counting methods, which have a higher risk of sampling bias than methods which involve a stereological approach (91); many histological variables can bias the results impacting on postmortem tissue such as longer fixation time, increased pH and post-mortem interval; psychiatric medication, non-prescription drugs and substance abuse (especially alcohol abuse) should be taken into account as possible confounding factors, especially because the lifetime exposure can alter brain biology. As far as psychotropic treatment in BD is concerned, both mood stabilizers and antipsychotics may drive promyelinating effects working on the inhibition of glycogen synthase kinase 3beta (GSK3β) throughout different biochemical pathways (38, 46). Lithium seems to mainly target subcortical WM throughout the GSK3β inhibition both directly via competition with magnesium and indirectly by increasing serine-phorphorylation of GSK3 through AKT (a serine/threonine kinase) activation (92), whereas first and second generation antipsychotics have been reported to promote predominantly intracortical myelination by inhibiting GSK3 signaling via dopamine 2 receptor blockade and 5HT2A receptor blockade (93).

Finally, oligodendrocyte alterations are not limited to BD but encompass most psychiatric conditions including major depression disorder, schizophrenia, attention-deficit and hyperactivity disorder and Alzheimer’s disease (94). The essential mechanism underling these alterations remains partially unknown; however, most of the current models point to a stress-related vulnerability mechanism prompted by disturbances in chromatin regulation, glutamate-mediated excitoxicity and glucocorticoid mechanism (95): a deficit/dysfunction in oligodendrocytes population could disrupt WM connectivity, affecting the trophic axonal support and thus determining a suboptimal conduction of action potentials.

## Conclusion

So far, DTI studies have consistently shown an alteration in prefrontal–limbic anatomical connectivity, leading to the hypothesis of BD as a connectivity disorder. Accordingly to this hypothesis, prefrontal regions would fail to regulate limbic regions, triggering emotional hyper reactivity and emotional instability. Besides this core and more consistent feature, rising lines of evidence are considering temporo-parietal WM abnormalities of long association tracts as a structural background of cognitive rather than affective symptoms in BD.

Although DTI studies provided compelling findings, some inaccuracies remain in the current DTI technique (18, 40, 68): clusters of WM alterations do not delineate tidily and WM tracts have an interindividual variability making it unclear which tracts are involved; WM microstructure may be affected by age, substance use, lithium use, antipsychotic use and genetic loading for BD; eventually, there are often differences among study populations with regard to bipolar subtype, affective state, duration of illness, medication status, comorbidities and family history.

Furthermore, morphological studies with larger and more homogeneous samples are required to clarify oligodendrocytes dysfunctions as well as to investigate the possible role of cortical and subcortical myelin plasticity in BD. In this regard, postmortem studies should have a key role to refine DTI studies’ results. Indeed, it should be clearly kept in mind that any inference on tissue microstructure arising from DTI data is based on an indirect measurement “prior to making bold claims about white matter integrity” (96).

## Author Contributions

MB and PB were responsible for conceptual design, manuscript, and table revision. FB was responsible for manuscript and table generation. ND and GV were responsible for main manuscript revisions. CA and MR were involved in the revision process.

## Conflict of Interest Statement

The authors declare that the research was conducted in the absence of any commercial or financial relationships that could be construed as a potential conflict of interest.

## Abbreviations

ACC, anterior cingulate cortex; CC, corpus callosum; DLPFC, dorsolateral prefrontal cortex; GSK3β, glycogen synthase kinase 3beta; IFOF, inferior fronto-occipital fasciculus; ILF, inferior longitudinal fasciculus; OD, oligodendrocyte density; OFC, orbitofrontal cortex; PFC, prefrontal cortex; sgACC, subgenual Anterior Cingulate Cortex; SLF, superior longitudinal fasciculus.
